# Whole-Genome Sequences of Phages p000v and p000y, Which Infect the Bacterial Pathogen Shiga-Toxigenic Escherichia coli

**DOI:** 10.1128/MRA.01400-18

**Published:** 2018-11-21

**Authors:** Cristina Howard-Varona, Dean R. Vik, Natalie E. Solonenko, M. Consuelo Gazitua, Zack Hobbs, Ryan W. Honaker, Anika A. Kinkhabwala, Matthew B. Sullivan

**Affiliations:** a Department of Microbiology, The Ohio State University, Columbus, Ohio, USA; b EpiBiome, Inc., Union City, California, USA; c Department of Civil, Environmental and Geodetic Engineering, The Ohio State University, Columbus, Ohio, USA; Loyola University Chicago

## Abstract

We report here the genome sequences and morphological characterizations of phages p000v and p000y, which infect the bacterial pathogen Shiga-toxigenic Escherichia coli O157:H7 and which are potential candidates for phage therapy against such pathogens.

## ANNOUNCEMENT

Antibiotic resistance in bacterial pathogens is growing at such an alarming rate throughout the world that the World Health Organization implores that researchers find alternatives to antibiotics to treat bacterial pathogens (1). One such solution is phage therapy, the use of bacteriophages against antibiotic-resistant bacteria (2). For Shiga-toxigenic Escherichia coli (STEC), which causes hemolytic uremic syndrome (3), antibiotic resistance is not only problematic but also can worsen the disease (4). Here, we present whole-genome sequences for two new phages that infect STEC O157:H7, p000v and p000y, as potential candidates for phage therapy against such pathogens.

**FIG 1:**
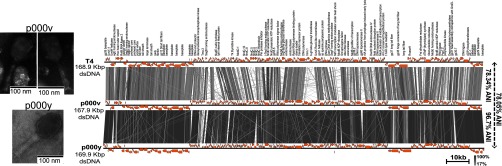
Morphological and genetic characterization of phages p000v and p000y. Whole-genome comparison of p000v, p000y, and E. coli phage T4 reveals that p000v and p000y are T4-like due to their high average nucleotide identity (ANI) shared with T4. For T4, GenBank sequence AF158101 was used. Shown via EasyFig 2.2.2 (10) are the percent protein similarities, obtained via a tblastx search (16) with an *e* value of 0.001, and their average nucleotide identities. Next to each genome is a transmission electron microscopy (TEM)-derived micrograph, which reveals that both phages have a Myoviridae morphology. TEM was performed at The Ohio State University (OSU) Campus Microscopy and Imaging Facility (CMIF) on an FEI Tecnai G2 Spirit TEM at an acceleration voltage of 80 kV, following a modified protocol (17). Samples were derived from 0.2-µm-filtered phage lysates that were concentrated with polyethylene glycol (p000v) and also purified with a CsCl (p000y) density gradient and dialyzed in phage buffer. For phage p000v, a 10-µl sample was deposited onto Formvar-coated 200-mesh copper TEM grids (Ted Pella, Inc., Redding, CA) and incubated for 30 min at room temperature. Grids were washed twice with distilled water and negatively stained with 10 µl of 2% uranyl acetate for 15 s. For p000y, a 200-µl sample was deposited onto the grids with an air-driven ultracentrifuge (Airfuge CLS, Beckman Coulter, Brea, CA), followed by negative staining as described.

Phages p000v and p000y were isolated from San Francisco, California, wastewater from overnight shaking cultures with exponentially growing laboratory E. coli cells in tryptone soya broth at 37°C, largely following previously described methods (5), and then isolated via the soft agar protocol (6, 7). The particles have Myoviridae morphology, as revealed by transmission electron microscopy (Fig. 1). Extraction and purification of genomic DNA was done from phage cultures using a phage DNA isolation kit (Norgen Biotek Corp., Thorold, ON, Canada) according to the manufacturer's protocol. The DNA library was prepared using the Nextera XT kit (Illumina, San Diego) according to the manufacturer's protocol (part number 15031942, revision D). The magnetic bead normalization step was replaced with a manual normalization step, based on library concentration and average size as measured by the Qubit 3.0 fluorometer and Qubit double-stranded DNA (dsDNA) high-sensitivity kit (Thermo Fisher Scientific, Waltham, MA) and the Fragment Analyzer (AATI, Ankeny, IA), respectively. Whole-phage DNA was sequenced with a MiSeq next-generation sequencing platform, using the MiSeq reagent v3 (600-cycle) kit (Illumina, San Diego, CA). A total of 100,000 paired reads of 300 bp without any quality trimming were assembled *de novo* using the SPAdes 3.9.1 genome assembler software using default parameters (8), with which one contig was obtained for each phage genome. The coverages for the genomes of phages p000v and p000y were 178.7× and 176.6×, respectively. Open reading frame prediction and annotation was done with the Prokka 1.12-beta software (9) and the *–kingdom viruses* option.

Phage p000v has a genome size of 167.9 kbp, with 37.6% GC content, and phage p000y has a genome size of 169.9 kbp and 37.7% GC content. Both phages share 96.7% identity, obtained via a whole-genome blastn 2.2.31 (10) analysis with an *e* value threshold of 0.0001. Additionally, both phages share >78% average nucleotide identity (ANI) with coliphage T4 (GenBank reference sequence AF158101) (11) (Fig. 1), obtained via the ANI calculator developed by Goris et al. (12). The shared genes included 38 T4-like phage core genes (defined by Sullivan et al. [13], with which a database was created) at >81% average identity, obtained via a blastn 2.2.31 (10) analysis. Both phages p000y and p000v appear to be T4-like myoviruses, based on conventional affiliation criteria (13, 14).

### Data availability.

Raw reads and assembled genome sequences of phages p000v and p000y are available in GenBank under the accession numbers MK047717 and MK047718, respectively, and on Cyverse (15) under DOI 10.7946/P2HP89 (https://www.doi.org/10.7946/P2HP89).
